# NOP2‐Mediated m5C Methylation Modification of LMNB2 mRNA Facilitates Colorectal Cancer Progression

**DOI:** 10.1002/cam4.70970

**Published:** 2025-05-14

**Authors:** Jinling Bi, Yong Huang, Wentao Hu, Yulong Liu

**Affiliations:** ^1^ Department of Oncology The Second People's Hospital of Hefei Hefei China; ^2^ Department of Oncology The Second Affiliated Hospital of Soochow University Suzhou China; ^3^ State Key Laboratory of Radiation Medicine and Protection, School of Radiation Medicine and Protection, Collaborative Innovation Center of Radiological Medicine of Jiangsu Higher Education Institutions Soochow University Suzhou China

**Keywords:** colorectal cancer, LMNB2, m5C, methyltransferase, NOP2

## Abstract

**Background:**

Colorectal cancer (CRC) is a leading cause of cancer‐related mortality globally, yet current therapies exhibit suboptimal efficacy with limited prognostic improvement. RNA 5‐methylcytosine (m5C), a posttranscriptional modification, has been implicated in tumorigenesis and progression across malignancies. In our previous study, the m5C methyltransferase NOP2 has been shown to promote proliferation, migration, and invasion of CRC cells, however, the underlying mechanism is still elusive.

**Methods:**

An integrated multi‐omics strategy was employed, combining transcriptomic sequencing, RNA immunoprecipitation sequencing (RIP‐seq), and methylated RNA immunoprecipitation sequencing (MeRIP‐seq) to explore NOP2‐regulated downstream genes mediating CRC progression via m5C methylation. Functional validation included in vitro and in vivo assays to assess tumor growth and metastasis. Rescue experiments were performed by overexpressing LMNB2 in NOP2‐silenced CRC cells.

**Results:**

NOP2‐dependent m5C modification of LMNB2 mRNA enhanced its stability, leading to elevated LMNB2 protein levels. This mechanism drove CRC tumor growth and metastasis both in vitro and in vivo. Overexpression of LMNB2 effectively rescued the suppressed malignant phenotypes induced by NOP2 knockdown, confirming LMNB2 as a critical downstream effector.

**Conclusion:**

NOP2 catalyzes the m5C modification of LMNB2 mRNA to facilitate its stability, which contributes to the elevated LMNB2 protein level and CRC progression, suggesting the potential of NOP2 as a therapeutic target in the development of novel CRC treatment.

## 1 Introduction

As one of the most common gastrointestinal malignancies in the world, colorectal cancer (CRC) has a high morbidity and mortality. According to the latest data acquired, the morbidity of CRC ranks third among all malignant tumors and the mortality rate ranks second [1]. With evolving dietary patterns accompanying rapid urbanization in developing countries, it is expected that the newly diagnosed cases worldwide would be 2.5 million annually by 2035. Besides, the incidence in young patients is becoming higher and higher [2]. The survival rate of patients with metastatic CRC is very low, and 20% of CRC patients have already experienced metastasis at the time of diagnosis [3]. Surgical resection has become the main therapeutic regimen, while radiotherapy and chemotherapy are often used as adjunctive therapies for surgical treatment, which exhibit high toxic effects while showing limited efficacy on patients with metastasis and recurrence. Molecular targeted therapy shows good efficacy and safety; however, the average effective rate of the currently available targeted drugs is only 15%–25% [4]. Therefore, clarifying the molecular mechanisms underlying the occurrence and development of CRC will be of great significance to the development of new therapeutic targets.

As a fundamental process in epigenetic regulation, RNA methylation is the most well‐characterized type of posttranscriptional modification of most types of RNA molecules. In recent years, significant achievements have been made in delineating the physiological and pathological functions of RNA methylation [5, 6, 7, 8]. Up to now, over 70 types of RNA methylations have been identified, including N6‐methyladenosine (m6A), N2‐methyladenosine (m2A), 7‐methylguanosine (m7G), N1‐methyladenosine (m1A), and 5‐methylcytosine (m5C), among which m6A is the most well‐known and common type, while there is relatively less research on m5C [9]. Recently, mounting evidence suggests that RNA m5C modification is involved in tumorigenesis and cancer progression [10]. Xu et al. found that the expression of m5C “reader” YBX1 is related to sorafenib resistance in hepatocellular cancer [11]. Wu et al. revealed that the m5C modification of circERI3 can increase its nuclear export, which in turn promotes the transcription of PGC‐1αthrough stabilizing DDB1 to affect mitochondrial function and energy metabolism, ultimately promoting the development of lung cancer [12]. Meanwhile, NSUN2‐mediated m5C modification of circFAM190B was reported to inhibit cellular autophagy through the SFN/mTOR/ULK1 pathway and ultimately promote lung cancer progression [13]. Zhao et al. found that NONO regulated the alternative splicing of PTEN mRNA in an m5C‐dependent manner, leading to decreased PTEN expression to facilitate gastric cancer progression [14]. Chen et al. found that m5C modification plays a crucial role in tumor microenvironment (TME) composition and complexity in CRC [15]. Besides, Yang et al. revealed that m5C modification of NXPH4 mRNA can avoid its degradation through RNA autophagy, thereby contributing to the malignant phenotypes of CRC via suppressing HIF1A degradation [16]. However, the regulatory mechanisms underlying the RNA m5C modification in CRC have not been fully understood.

In a previous study, we found that proliferation‐associated nucleolar protein p120 (NOP2), which is one of the highly conserved eukaryotic m5C methyltransferases, is significantly upregulated in colon cancer tissues and cells compared with that in the healthy controls, functioning as an oncogene that promotes proliferation, migration, and invasion of CRC cells [17]. However, the underlying mechanism is still elusive. In this study, we carried out an integrated multi‐omics strategy combining transcriptome sequencing, RNA immunoprecipitation sequencing (RIP‐seq), and m5C‐RIP sequencing to explore the molecular mechanism of NOP2‐mediated epigenetic regulation in the progression of CRC.

## 2 Materials and Methods

### 2.1 Cell Culture and Transfection

HCT116, NCM460, SW480, HT29, and LoVo cells were purchased from the National Collection of Authenticated Cell Cultures and cultured in DMEM medium (Sigma, St. Louis, MO, USA) supplemented with 10% FBS (Gibco, Grand Island, NY, USA), 1% penicillin sodium, and 100 μg/mL streptomycin at 37°C in a humidified incubator containing 5% CO_2_. The LMNB2 overexpression vectors, siNC, and siNOP2 were designed and synthesized by RiboBio (Guangzhou, China) and transfected into cells by using Lipofectamine 3000 Reagent (Invitrogen, Carlsbad, CA, USA) under the guidance of the manufacturer's introduction.

### 2.2 qRT‐PCR

RNA isolation reagent (Vazyme, Nanjing, China) was used to extract total RNA from cells and tissues. Transcription of mRNA was performed with Prime Script RT kit (Vazyme, Nanjing, China). For the polymerase chain reaction, the AceQ qPCR SYBR Green Master Mix (low ROX premixed) (Vazyme, Nanjing, China) was used. The Vii7A system (Thermo Fisher Scientific, Waltham, MA, USA) was used for quantitative PCR and signal generation. PCR data were treated with the 2^−△△Ct^ method to analyze the relative expression of genes. GAPDH was used as the internal reference gene, and the primer sequences used for PCR are listed in Table 1.

**TABLE 1:** Oligonucleotide primer sequences used for qPCR.

Gene	Sequence
NOP2 forward	5′‐TGTCTGAGCTGGTGGAGTTCTTAG‐3′
NOP2 reverse	5′‐ACCCCACGATTGATTAGAGCC‐3′
LMNB2 forward	5′‐GCAGAGTTGGACGAGGTCAA‐3′
LMNB2 reverse	5′‐GCTTTTTGGCCACTGCATGA‐3′
GAPDH forward	5′‐AGAAGGCTGGGGCTCATT‐3′
GAPDH reverse	5′‐TGCTAAGCAGTTGGTGGTG‐3′

### 2.3 Wound Healing Assay

Cells were cultured in 6‐well plates at a density of 1 × 10^5^ per well until the cell confluency reached about 90% of the well. The spent culture medium was changed to fresh medium containing 1% FBS, and the cells were treated for 12 h. Then, the wound was made with a 200 μL pipette tip, and the cells were washed several times with PBS to discard the floating cells. The images of each wound were taken at 0 and 24 h after the wound was made, and Image‐J software (version 1.8.0, National Institutes of Health, Bethesda, MD, USA) was used for wound area analysis.

### 2.4 Transwell Assay

Cells were seeded in 6‐well plates at a density of 1 × 10^5^ per well and cultured in mediums containing 1% FBS for 12 h. The pre‐cooled 8 μm transwell chambers were filled with 60 μL Matrigel (Corning, Corning, NY, USA) per well, which solidified at 37°C for 30 min. Then, cells were digested with trypsin and seeded in a transwell chamber at a density of 3 × 10^4^ per well. After 24 h, the chamber was washed with PBS several times and fixed with 75% alcohol for 10 min at room temperature. Next, the fixed cells were stained with 0.1% crystal violet for 10 min at room temperature, and the cells in the top chamber were removed slightly. Then, photos of the cells in the bottom chamber were taken with a Leica microscope, and the cell number was analyzed with Image‐J software.

### 2.5 Western Blotting

The total protein of cells was collected by using RIPA lysis buffer (Beyotime, Shanghai, China) and processed into protein samples by using protein loading buffer after concentration determination using a DC Protein Assay Kit (Bio‐Rad, Richmond, CA, USA). The protein samples were then separated by SDS‐PAGE system and transferred to PVDF membranes (Amersham, Arlington, IL, USA). After blocking, first‐antibody incubation, and secondary antibody incubation, the chemiluminescent signal was acquired using an ECL kit (Millipore, Bedford, MA, USA) and a polychromatic fluorescence chemiluminescence imaging analysis system (Alpha, San Leandro, CA, USA). Antibodies used in the above experiments were NOP2 (10448‐1‐AP, Proteintech, Chicago, IL, USA), GAPDH (10494‐1‐AP, Proteintech, Chicago, IL, USA), LMNB2 (ab151735, Abcam, Cambridge, MA, USA), and β‐Actin (20536‐1‐AP, Proteintech, Chicago, IL, USA).

### 2.6 Methylated RNA Immunoprecipitation and Quantitative PCR (MeRIP‐qPCR)

Cells were transfected with siNC or siNOP2. After treatment, the total RNA of the cells was isolated. The RNA samples were treated with a m5C MeRIP kit (Cloudseq Biotech, Shanghai, China) according to the manufacturer's introduction to acquire m5C RNA immunoprecipitation (m5C‐RIP) products. The m5C‐RIP products were performed with methylation‐specific PCR by using methylation‐specific primers targeting the m5C modification peak of LMNB2 mRNA. The sequences of primers used in the experiments were listed as follows: LMNB2‐M Forward: 5′‐GAGGAGAGGTTGAAGTTGTTTTTTA‐3′; LMNB2‐M Reverse: 5′‐AACTACACAAACTTACCCTCCAAAT‐3′.

### 2.7 Cell Proliferation

For the cell counting assay, cells were seeded in 12‐well plates at a density of 1 × 10^5^ per well. Then the cells were transfected with siNC or siNOP2. At 24, 48, 72, and 96 h after transfection, the cells were digested and counted to acquire the cell number of each time point. Each group has three parallel wells. For the EdU staining assay, cells were seeded in 6‐well plates that had pre‐put 13 mm circular glass slides in the wells. Then siNC, siNOP2, and LMNB2 overexpression vectors were transfected into cells. After 24 h, the cells were stained with EdU (BeyoClick EdU‐647, Beyotime, Shanghai, China) according to the manufacturer's instructions. Then the cells were stained with DAPI and processed into sections. The images of the cells were taken by a laser scanning confocal microscope (Olympus, Tokyo, Japan) and analyzed by Image‐J software.

### 2.8 Apoptosis

HCT116 and LoVo cells were seeded in 6‐well plates at a density of 1 × 10^5^ per well. Then the cells were transfected with siNC, siNOP2, and LMNB2 overexpression plasmids. After 24 h, cells in each well were collected, and the Annexin V‐Alexa Fluor 647–Propidium Iodide Apoptosis Detection kit (FCMACS Biotech, Nanjing, China) was used for the Annexin V and Propidium Iodide staining. Then the cells were measured by flow cytometry (BD Bioscience, San Jose, CA, USA), and the apoptosis rate of the cells was determined.

### 2.9 Animal Experiment

Shanghai SLACCAS Animal laboratory (Shanghai, China) provided male BALB/c nude mice aged 6–8 weeks, and a specific pathogen‐free environment was used for mice feeding. The animal experiment protocol for this study was approved by the Laboratory Animal Ethics Committee of Soochow University.

For subcutaneous tumor model construction, 5 × 10^6^ HCT116 cells were subcutaneously injected into the left and right lower flanks of each mouse. When tumor volume reached about 100 mm^3^, animal‐used siRNA targeting NOP2 (siNOP2) and negative control siRNA (siNC) (RiboBio, Guangzhou, China) were injected into the solid tumors every 3 days with a dose of 1 nmol/time. The tumor length (*a*) and width (*b*) were measured every 3 days, and the tumor volume was calculated by the formula: *V* = *ab* ^2^ × 0.52. After 21 days of treatment and measurement, all mice were sacrificed, and the subcutaneous solid tumors were harvested and weighed.

For metastasis model construction, Luciferase‐HCT116 cells transfected with siNC or siNOP2 were injected into each mouse through the tail vein with 5 × 10^6^ cells per mouse. After 1 month, 100 μL D‐luciferin potassium salt (2 mg/mL) (Beyotime, Shanghai, China) was injected into the mouse abdominal cavity, and then the IVIS Spectrum In Vivo Imaging System (PerkinElmer, Hopkinton, MA, USA) was used to acquire the fluorescence signal of the mouse body.

### 2.10 Immunohistochemistry (IHC)

The tumor tissues were fixed after harvest and embedded in paraffin, then the embedded tissues were processed into sections. For immunohistochemistry, the sections were incubated with NOP2 (10448‐1‐AP, Proteintech, Chicago, IL, USA), LMNB2 (ab151735, Abcam, Cambridge, MA, USA), or Ki‐67 (34330, CST, Danvers, MA, USA) antibodies at 4°C overnight. Then the sections were washed and incubated with secondary antibodies (PV‐6001, PV‐6002, ZSGB‐Bio, Beijing, China) at 37°C for 30 min. After antibody incubation, the sections were treated with DAB agent (ZLI‐9019, ZSGB‐Bio, Beijing, China) and sealed in neutral balsam. The photos of the sections were acquired by using an inverted microscope (Olympus, Tokyo, Japan) and analyzed by Image‐J software to quantify the positive rate of the target genes.

### 2.11 Patients Samples

The clinical CRC samples were collected from the Second People's Hospital of Hefei (Anhui, China) after surgery between 2018 and 2023. None of the CRC patients had received radiotherapy or chemotherapy before surgery. The 26 pairs of normal colorectal tissues and CRC tissues were used to extract RNA to test NOP2 and LMNB2 mRNA expression. The pathological diagnosis was confirmed by two clinically experienced pathologists. The Research Ethics Committee of the Second People's Hospital of Hefei approved the study, and all patients provided signed informed consent. The patients' information is listed in Table S1.

### 2.12 Dual‐Luciferase Reporter Assay

The LMNB2 mRNA coding sequence (CDS) including the m5C sites and the corresponding mutant sequence was constructed into the pmirGLO reporter vector (RiboBio, Guangzhou, China) respectively. Lipofectamine 3000 reagent was used for luciferase vector transfection into siNC and siNOP2‐treated cells. Dual‐Luciferase Reporter Assay kit (Beyotime, Shanghai, China) was used for luciferase activity detection of transfected cells according to the manufacturer's instructions.

### 2.13 Statistical Analysis

The statistical analysis of data was performed by GraphPad Prism 8.0 and SPSS 16.0 software. The difference between the two groups was analyzed by Student's *t*‐test, and three or more group comparisons were analyzed by ANOVA. Data are presented as mean ± standard deviation (SD). The *p*‐value less than 0.05 was considered a statistical difference.

## 3 Results

### 3.1 NOP2 is Highly Expressed in CRC

**FIGURE 1:**
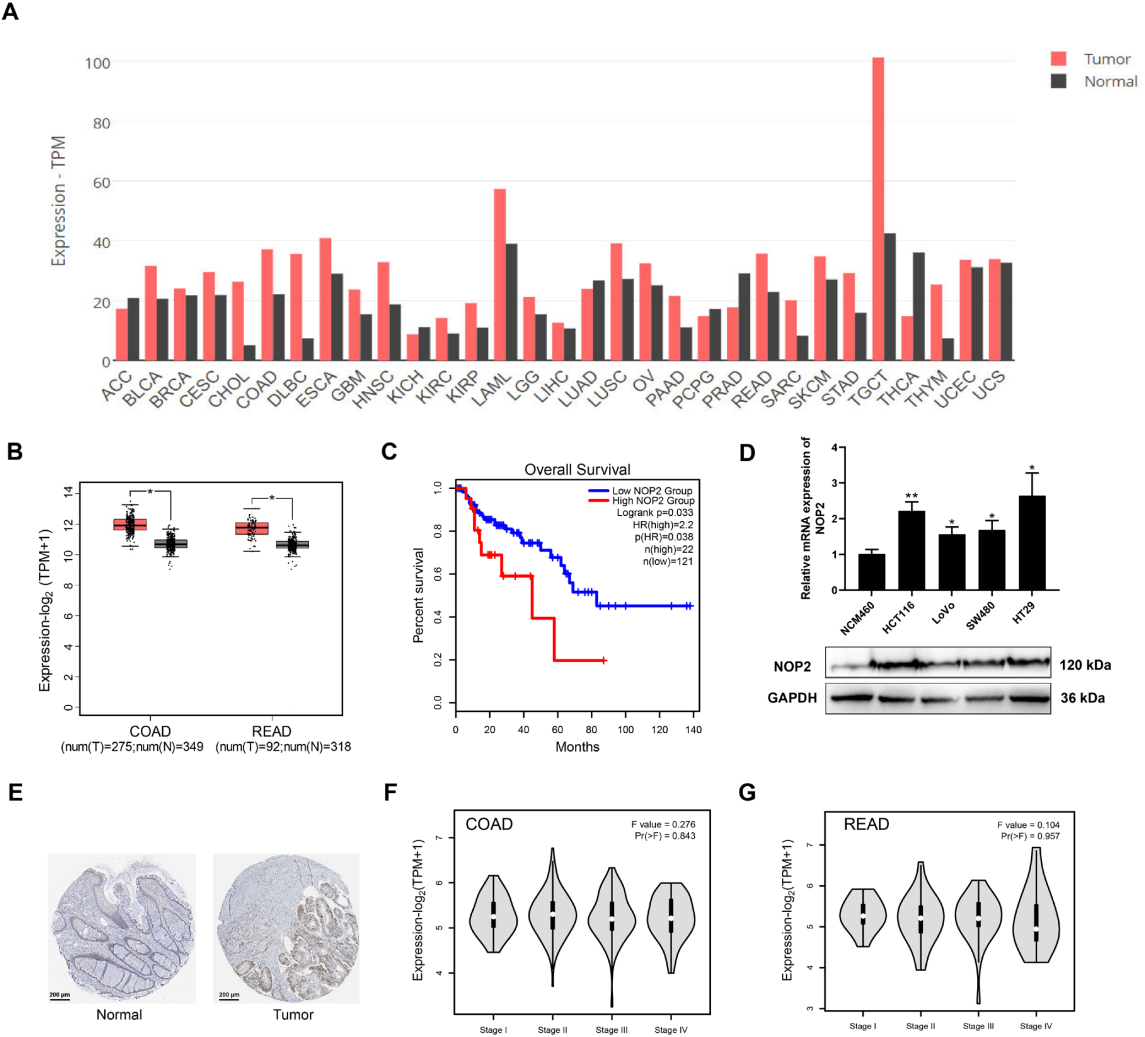
NOP2 is highly expressed in CRC tissues and cell lines. (A) NOP2 expression in different types of cancers and the adjuvant normal tissues. (From GEPIA2 database). (B) Relative expression of NOP2 in COAD and READ tumor tissues compared with adjacent normal tissues was analyzed in the GEPIA2 database. (C) The survival curve of CRC patients with high or low NOP2 expression levels was predicted in the OncLnc database. (D) The relative mRNA expressions and protein levels of NOP2 in four different CRC cell lines and one normal colon cell line (NCM460). (E) Relative expression of NOP2 in CRC tumor tissues and adjuvant normal tissues. Data from the HPA database. (F, G) NOP2 expression in different stages of COAD or READ (from GEPIA2). All data were exhibited with mean ± SD (*n* = 3). *p < 0.05; **p < 0.01.

NOP2 has been reported to participate in m5C methylation of mRNA and is associated with CRC progression. Through exploring the GEPIA2 database, we found high expression of NOP2 in various kinds of cancers (Figure 1A). Here, to further confirm the function of NOP2 for CRC, we detected the NOP2 expression in COAD and READ tumor tissues and adjacent normal tissues. As shown in Figure 1B, GEPIA2 database analysis results show that COAD and READ tumor tissues have higher NOP2 levels than normal tissues. Figure 1C shows that patients with higher NOP2 levels exhibited poorer prognoses, indicating the function of NOP2 as an oncogene. Besides, we also measured the NOP2 mRNA expressions and protein levels in CRC cell lines; the results showed that CRC cells (HCT116, LoVo, SW480, and HT29) have higher NOP2 levels than normal cells (NCM460) (Figure 1D). Besides, we found that CRC tissues have higher NOP2 protein expressions than normal tissues through the data from the HPA database (Figure 1E). Further investigation shows that the NOP2 expressions in different stages of COAD and READ do not present a significant difference (Figure 1F,G). All these data suggest that NOP2 is highly expressed in CRC tissues and cell lines.

### 3.2 Silencing of NOP2 Inhibits the Migration, Invasion, and Proliferation of CRC Cells

**FIGURE 2:**
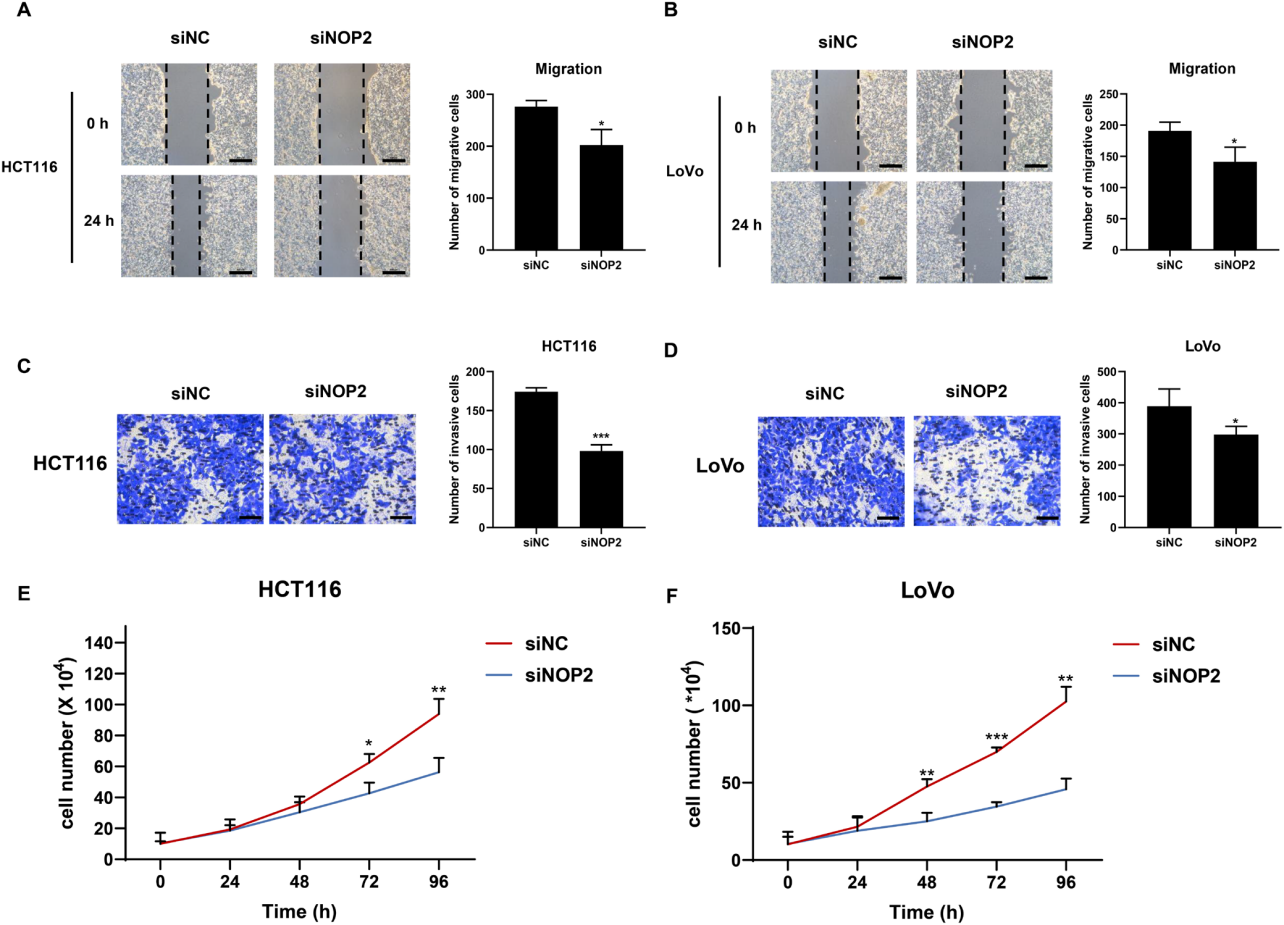
Silencing of NOP2 inhibited the proliferation, invasion, and migration of CRC cells. (A, B) The migration ability of siNC or siNOP2‐transfected HCT116 and LoVo cells was detected by wound healing assay. Scale bar = 400 μm. (C, D) The invasion ability of HCT116 and LoVo cells was detected by transwell assay with Matrigel in transwell chambers. Scale bar = 50 μm. (E, F) The proliferation of HCT116 or LoVo cells was detected through cell number counting at different time points after transfecting siNC or siNOP2. All data were exhibited with mean ± SD (*n* = 3). *p < 0.05; **p < 0.01; ***p < 0.001.

To explore the effects of NOP2 on migration, invasion, and proliferation of CRC cells, we transfected siRNA targeting NOP2 (siNOP2) and negative control siRNA (siNC) into HCT116 and LoVo cells. As shown in Figure 2A, the migration and invasion ability of HCT116 and LoVo cells were all significantly decreased in the siNOP2 group. The proliferation ability of the two cell lines was also inhibited by the silencing of NOP2 (Figure 2E,F). The above data suggested that NOP2 may play an important regulatory role in CRC cell growth and mobility process.

### 3.3 LMNB2 is Positively Regulated by NOP2 in CRC

**FIGURE 3:**
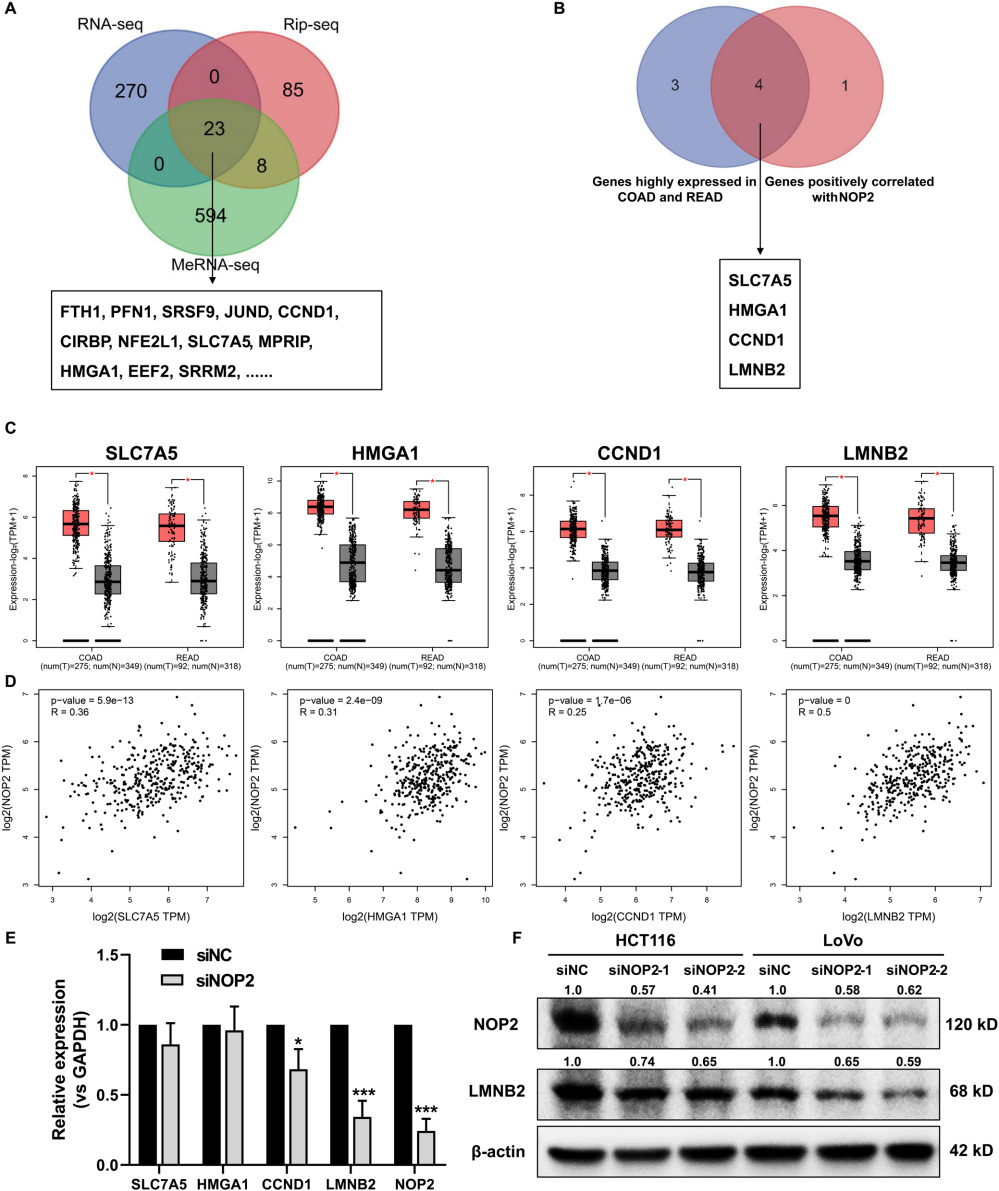
Silencing of NOP2 induced the downregulation of LMNB2. (A) HCT116 cells were transfected with siNC or siNOP2, then multi‐omics sequencing was performed. The differentially expressed genes were intersected, and 23 significantly downregulated genes regulated by siNOP2 were selected for further analysis. (B) The 23 selected genes were analyzed in the GEPIA2 database, and 4 genes highly expressed in COAD and READ and positively correlated with NOP2 were selected. (C) The relative expressions of SLC7A5, HMGA1, CCND1, and LMNB2 in COAD and READ tumor tissues compared with adjuvant normal tissues were from the GEPIA2 database. (D) The co‐expression analysis between NOP2 and SLC7A5, HMGA1, CCND1, or LMNB2 in COAD and READ was performed in the GEPIA2 database. (E) After silencing NOP2, the expression of SLC7A5, HMGA1, CCND1, and LMNB2 was measured through qRT‐PCR. (F) NOP2 and LMNB2 protein levels in NOP2‐silenced HCT116 and LoVo cells. All data were exhibited with mean ± SD (*n* = 3). *p < 0.05; ***p < 0.001.

Based on the methyltransferase function of NOP2, the gene methylation level regulated by NOP2 may play an essential role in promoting CRC progression. Next, to investigate the potential downstream genes that are possibly regulated by NOP2, we performed RNA sequencing, RNA immunoprecipitation sequencing (RIP sequencing), and methylated RNA immunoprecipitation sequencing (MeRIP‐seq) for siNC and siNOP2‐transfected HCT116 cells. As shown in Figure 3A, 23 genes were selected for further analysis. Next, we predicted these 23 genes in the GEPIA2 database to find genes that are highly expressed in COAD and READ and positively related to NOP2 expression, and four genes were selected (Figure 3B–D). Besides, we detected the mRNA expression of these four genes in NOP2‐silenced cells, and the results indicated that the knockdown of NOP2 induced the downregulation of CCND1 and LMNB2, and LMNB2 was more significantly decreased (*p* = 0.0006) (Figure 3E). Consistently, the downregulation of LMNB2 protein level in NOP2‐silenced HCT116 and LoVo cells also led to a decreased protein level of LMNB2 (Figure 3F). All these data suggested that LMNB2 may be positively regulated by NOP2.

### 3.4 NOP2 Promotes the Methylation of LMNB2 mRNA in CRC

**FIGURE 4:**
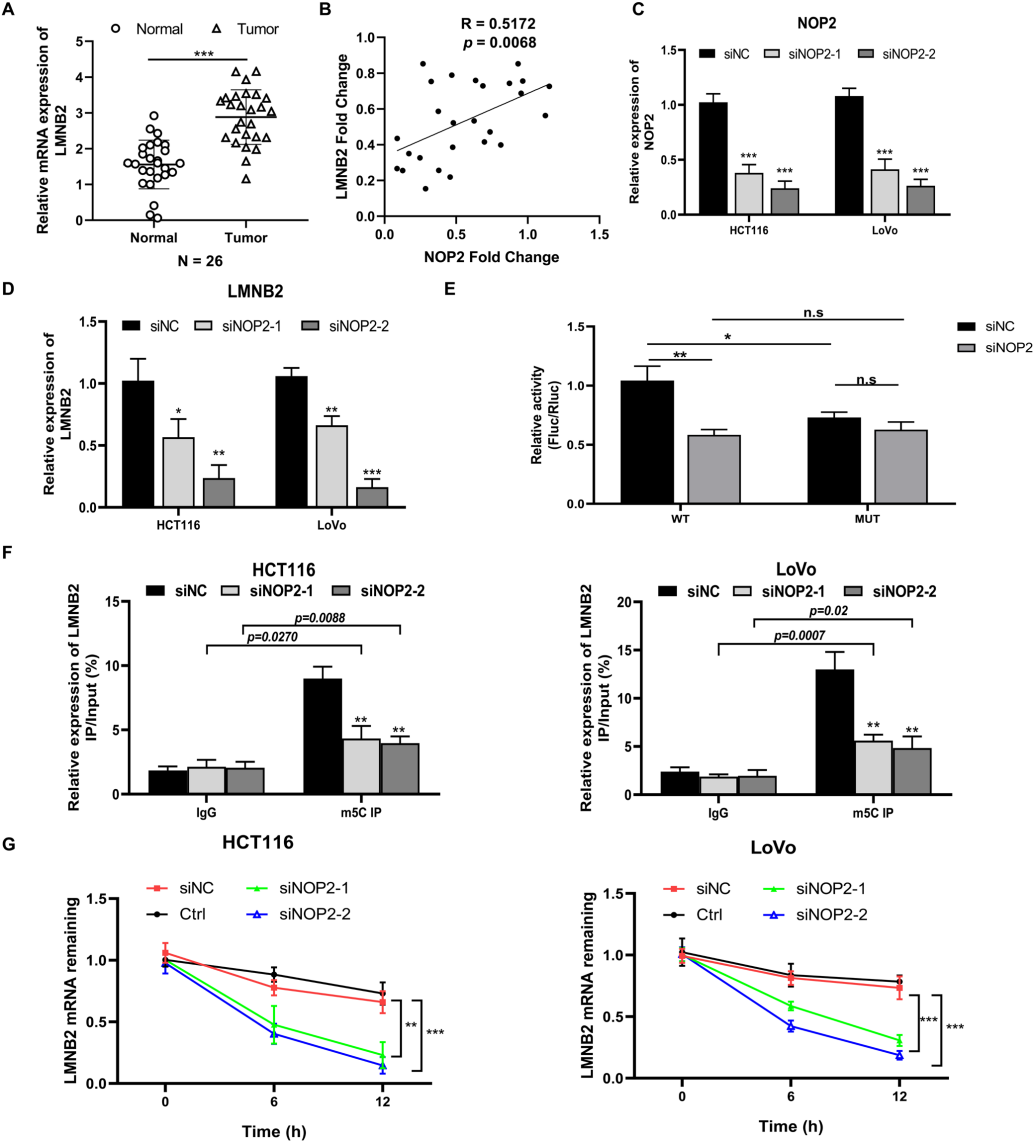
NOP2 promotes the m5C methylation of LMNB2 mRNA. (A) The mRNA expression of LMNB2 in 26 pairs of CRC tumor tissues and adjuvant normal tissues was detected by qRT‐PCR. (B) Co‐expression analysis between NOP2 and LMNB2 in 26 CRC tumor tissues. (C) The relative NOP2 expression in NOP2‐silenced HCT116 and LoVo cells. (D) LMNB2 expression in NOP2‐silenced HCT116 or LoVo cells. (E) Dual‐luciferase assay was used to test the relative luciferase activity of a luciferase reporter with WT (wild‐type) or MUT (mutant) LMNB2 CDS sequence containing the m5C site in NOP2 siRNA‐ or siNC‐transfected HCT116 cells. (F) The LMNB2 mRNA methylation levels in NOP2‐silenced cells were detected by MeRIP‐qPCR. (G) The LMNB2 mRNA stability in HCT116 and LoVo cells with NOP2 knockdown. All data were exhibited with mean ± SD (*n* = 3). *p < 0.05; **p < 0.01; ***p < 0.001.

In the 26 pairs of clinical CRC samples, we measured the expression of LMNB2 and confirmed the higher LMNB2 level in tumor samples than in normal samples (Figure 4A). As shown in Figure 4B, the co‐expression analysis results show that the expression of NOP2 is positively related to the expression of LMNB2 (*R* = 0.5172, *p* = 0.0068). In addition, we confirmed the LMNB2 mRNA expression was inhibited in NOP2‐silenced HCT116 and LoVo cells (Figure 4C,D). Considering the methylation modification function of NOP2, we performed MeRIP‐seq to explore m5C modification changes in mRNA after silencing NOP2, and Figure S1 shows the most common motif of these enrichment peaks was CHDCCACC in the siNC group and AGGARGAA in the siNOP2 group, respectively. As predicted by RNAm5Cfinder, the coding sequence region (CDS) of LMNB2 mRNA contains the m5C methylation sites (Figure S2). Thus, we conducted a luciferase reporter assay to verify the predicted m5C site. As shown in Figure 4E, the luciferase activity was significantly decreased in NOP2‐silenced cells transfected with the wild‐type construct, while no significant difference in the luciferase activity was detected with mutant vectors. Furthermore, we detected the LMNB2 mRNA methylation level through MeRIP‐qPCR. As shown in Figure 4F, silencing of NOP2 significantly decreased the methylation level of LMNB2 mRNA. The LMNB2 mRNA stability was also obviously decreased in HCT116 and LoVo cells with NOP2 knockdown (Figure 4G). These data indicated that downregulation of NOP2 decreased both the methylation level and stability of LMNB2 mRNA and further inhibited the LMNB2 expression.

### 3.5 Overexpression of LMNB2 Can Rescue the Suppressed Malignant Phenotypes Caused by NOP2 Knockdown

**FIGURE 5:**
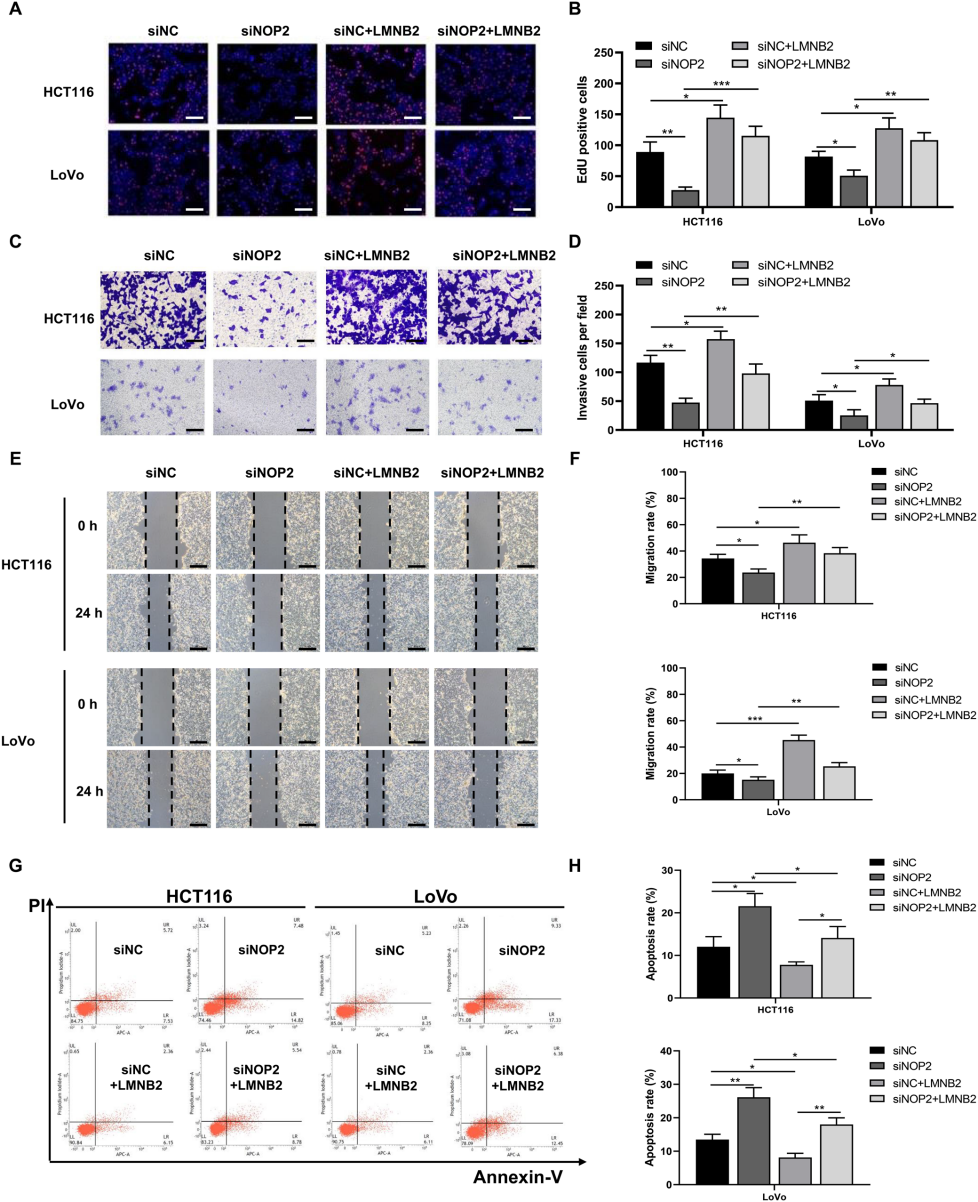
LMNB2 promotes the proliferation, invasion, and migration abilities of HCT116 and LoVo cells. HCT116 and LoVo cells were transfected with siNOP2 or LMNB2 overexpression vectors. (A, B) EdU staining was performed to measure cells' proliferation. Blue immunofluorescence indicated nuclear and red indicated EdU‐positive cells. The EdU‐positive cells in per field were also quantified. Scale bar = 50 μm. (C, D) The invasion ability of cells was measured by transwell assay, and the number of invasive cells in per field was quantified. Scale bar = 50 μm. (E, F) The migration ability of cells was measured by the wound healing assay and the migration rate was quantified. Scale bar = 400 μm. (G, H) Apoptosis level of HCT116 and LoVo cells was measured by flow cytometry and cells apoptosis rate was quantified. All data were exhibited with mean ± SD (*n* = 3). *p < 0.05; **p < 0.01; ***p < 0.001.

To explore the cellular function of LMNB2 in CRC cells, we transfected siNOP2 or LMNB2 overexpression vectors into HCT116 and LoVo cells. Then we performed cellular proliferation, migration, and invasion assays for HCT116 and LoVo cells. EdU staining results showed that silencing of NOP2 significantly decreased the number of EdU‐positive cells, while overexpression of LMNB2 can rescue the inhibition effect (Figure 5A,B). Similarly, the downregulation of NOP2 remarkably decreased the number of invasive cells and migration rate of both HCT116 and LoVo cells, while the upregulation of LMNB2 can reverse these effects (Figure 5C–F). In addition, we further tested the CRC cells' apoptosis rate after transfecting siNOP2 and LMNB2 overexpression vectors. The results showed that the knockdown of NOP2 promoted the apoptosis rate of both CRC cell lines, while the overexpression of LMNB2 significantly decreased the apoptosis rate (Figure 5G,H). All the above data suggested that NOP2 and LMNB2 play a promotional function for CRC cells' proliferation, invasion, and migration ability. The overexpression of LMNB2 can rescue the suppressed malignant phenotypes caused by NOP2 knockdown.

### 3.6 Silencing of NOP2 Inhibited the Progression of CRC In Vivo

**FIGURE 6:**
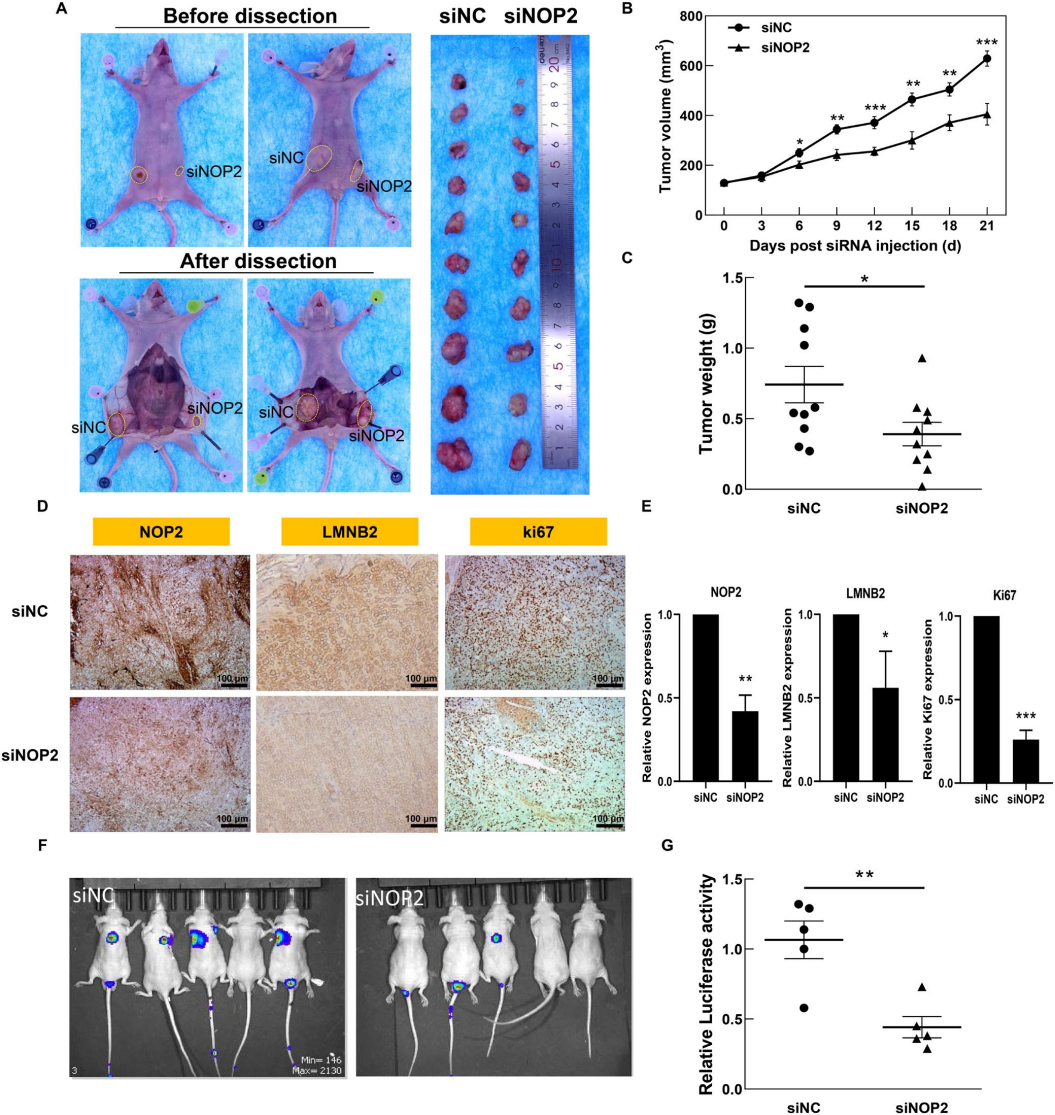
Silencing of NOP2 inhibited the growth and metastasis of CRC tumors and downregulated the expression of LMNB2 in HCT116‐derived xenografts. (A) The image of dissected tumor tissues. (B) Tumor growth curve during 21 days of treatment. (C) Weight of the dissected tumor tissues. (D, E) IHC images of tumor tissues stained with NOP2, LMNB2, or Ki67, and the relative expression of these genes was quantified by calculating the positive area. (F, G) The images of mice that were injected with HCT116 cells through tail vein injection. The signal intensity of each mouse was quantified. Scale bar = 100 μm. All data were exhibited as mean ± SD (*n* = 3). *p < 0.05; **p < 0.01; ***p < 0.001.

In previous work, we confirmed the oncogenic function of NOP2 in vitro. To further verify the obtained results in vivo, we first constructed the CRC tumor model by subcutaneously injecting HCT116 cells into nude mice. When the tumor volumes reached about 100 mm^3^, we performed eight rounds of intra‐tumor injection of siNC or siNOP2. As shown in Figure 6A–C, the silencing of NOP2 significantly decreased the tumor growth, tumor volume, and tumor weight during 21 days of treatment. The IHC results also show that expressions of NOP2, LMNB2, and Ki67 were all decreased in the siNOP2 group, compared with the siNC group (Figure 6F,G). Furthermore, we constructed a CRC metastasis mouse model through tail vein injection of luciferase‐marked HCT116 cells with or without NOP2 knockdown. The results indicated that the downregulation of NOP2 significantly inhibited the lung metastasis rate of HCT116 cells (Figure 6D,E). All these data suggested that the knockdown of NOP2 obviously inhibits both growth and metastasis of HCT116‐derived xenografts in vivo, indicating that NOP2 holds the potential to be used as a therapeutic target in adjuvant CRC therapy.

## 4 Discussion

CRC remains one of the most common gastrointestinal malignancies all over the world, with a high incidence rate and mortality [1]. At present, surgery and chemotherapy are still the main treatments for CRC, and the limitations of molecular targeted therapy are still significant. Therefore, it is of great significance to conduct in‐depth research on the biological mechanisms underlying the occurrence and development of CRC, and to search for effective molecular targets for the treatment of CRC. Methyltransferase NOP2, also known as NOL1, NSUN1, or p120, catalyzes the m5C modification of 28S rRNA and mRNA [18, 19, 20]. Previous research has shown that the expression of NOP2 is related to the malignancy of CRC [17]. However, the underlying molecular mechanism remains unclear.

In this work, we found that the CRC highly expressed NOP2, which was reported to be associated with the methylation of mRNA. To further explore the role of NOP2‐mediated RNA methylation in CRC, we performed integrative multi‐omics analyses (RNA‐seq, RIP‐seq, and MeRIP‐seq) for NOP2‐silenced CRC cells (HCT116). The intersection analysis for three sequencing data sets shows that 23 genes were both downregulated and demethylated by the knockdown of NOP2. Among them, four candidate genes that are positively correlated with NOP2 expression and also predict the poor prognosis of CRC patients were selected. Further experiments suggested that LMNB2 mRNA exhibited the most significant downregulation upon NOP2 knockdown. Meanwhile, it was suggested that the methylation level of LMNB2 mRNA was significantly decreased, contributing to the decreased mRNA stability. These data together illustrate that NOP2 catalyzes the methylation modification of LMNB2 mRNA to stabilize it and positively regulates the expression of LMNB2. Besides, if we knocked down NOP2, the LMNB2 mRNA stability was also decreased, indicating that the LMNB2 expression was positively regulated by the methylation modification of LMNB2 mRNA. Then, cell function experiment results show that the cell proliferation, migration, and invasion abilities of HCT116 and LoVo cells were inhibited significantly in the NOP2 knockdown group, while the overexpression of LMNB2 rescued the inhibited phenotypes. The increased cell apoptosis rate induced by NOP2 silencing was also rescued by LMNB2 overexpression. These data indicated that NOP2 regulates CRC progression by promoting LMNB2 expression. In vivo experiments also confirmed that silencing of NOP2 inhibited tumor growth, metastasis, as well as LMNB2 expression.

LMNB2 has been reported to be able to promote tumorigenesis, growth, and metastasis across a variety of tumors in several studies [21, 22, 23, 24]. LMNB2 promotes CRC progression by silencing p21 expression and facilitating cell cycle progression. In lung adenocarcinoma, KLF16 enhances the transcription activity of LMNB2 and promotes lung cancer cell growth and invasion [25]. In the central nervous system, elevated expression of LMNB2 is associated with the rapid progression of glioma. Overexpression of LMNB2 significantly induced nuclear morphological aberrations in normal human astrocytes and accelerated cell proliferation [23]. Besides, it was also revealed that LMNB2 expression was upregulated in human bladder cancer tissues, and it promoted the proliferation of bladder cancer cells via transcriptional activation of CDCA3 expression [26]. In this study, we found that the expression of LMNB2 is higher in CRC tissues compared with the adjacent normal tissues. The LMNB2 upregulation promoted the viability, migration, and invasion of CRC cells, while inhibiting their apoptosis. Besides, we demonstrated the stabilization of LMNB2 mRNA via NOP2‐mediated m5C methylation. Our results indicated that LMNB2 plays a role in promoting CRC progression in a NOP2‐dependent m5C modification. The translational significance of our findings is underscored by the targeted therapy potential of NOP2 inhibition. The observed NOP2/LMNB2 axis suggests a potential novel therapeutic axis for overcoming chemoresistance in CRC, which warrants validation in future patient‐derived CRC organoid studies.

While our study establishes the NOP2‐LMNB2 axis as a key driver of CRC progression, several questions merit further investigations. Although our study establishes m5C methylation as the primary mechanism for LMNB2 regulation, future work should explore combinatorial effects of m5C with other epigenetic modifications (e.g., m6A/m1A) and non‐coding RNA networks. Besides, the crosstalk between NOP2‐dependent LMNB2 stabilization and known CRC drivers (e.g., APC/β‐catenin pathway) represents an important direction for future studies. Furthermore, due to the limited sample size in our study, the correlation between NOP2 expression levels and clinicopathological characteristics needs validation in larger patient cohorts in future studies.

## 5 Conclusions

Based on our results, we concluded that NOP2 facilitates CRC progression by enhancing the m5C methylation modification of LMNB2 mRNA. This study provides the first experimental evidence revealing that NOP2‐dependent m5C modification of LMNB2 mRNA increases the LMNB2 protein expression by enhancing its mRNA stability, thereby facilitating CRC tumor growth and metastasis. These results provided new evidence for clarifying the molecular mechanism of NOP2 in promoting CRC progression, holding promise for developing m5C‐targeted therapies.

## Author Contributions

Conceptualization, J.B., Y.L.; methodology, Y.H.; software; validation, Y.H.; formal analysis, J.B.; investigation, J.B., Y.H.; data curation, J.B., Y.H.; writing – original draft preparation, J.B.; writing – review and editing, W.H., Y.L.; visualization, J.B.; supervision, W.H., Y.L.; project administration and funding acquisition, J.B., W.H., and Y.L. All authors have read and agreed to the published version of the manuscript.

## Ethics Statement

The study was conducted in accordance with the Declaration of Helsinki and approved by the Ethics Committee of Soochow University.

## Consent

Informed consent was obtained from all subjects involved in the study.

## Conflicts of Interest

The authors declare no conflicts of interest.

## Supporting information


**Figure S1:** The differentially changed m5C modification peaks and the peaks distribution between siNC and siNOP2 groups.
**Figure S2:** The m5C modification sites in the CDS region of LMNB2 mRNA were predicted by the online tool RNAm5Cfinder.
**Table S1:** Information of deep‐frozen CRC specimens.

## Data Availability

Data is available upon request to the corresponding author.
