# Supplementary material for: NOP2‐Mediated m5C Methylation Modification of LMNB2 mRNA Facilitates Colorectal Cancer Progression
Source: Cancer Med. 2025 May 14;14(10):e70970. doi: 10.1002/cam4.70970 (PMC12076344; doi:10.1002/cam4.70970)
Supplement: Supplementary file 1 — Figure S1: The differentially changed m5C modification peaks and the peaks distribution between siNC and siNOP2 groups. Figure S2: The m5C modification sites in the CDS region of LMNB2 mRNA were predicted by the online tool RNAm5Cfinder. Table S1: Information of deep‐frozen CRC specimens. [file CAM4-14-e70970-s001.docx]

Supplementary Material


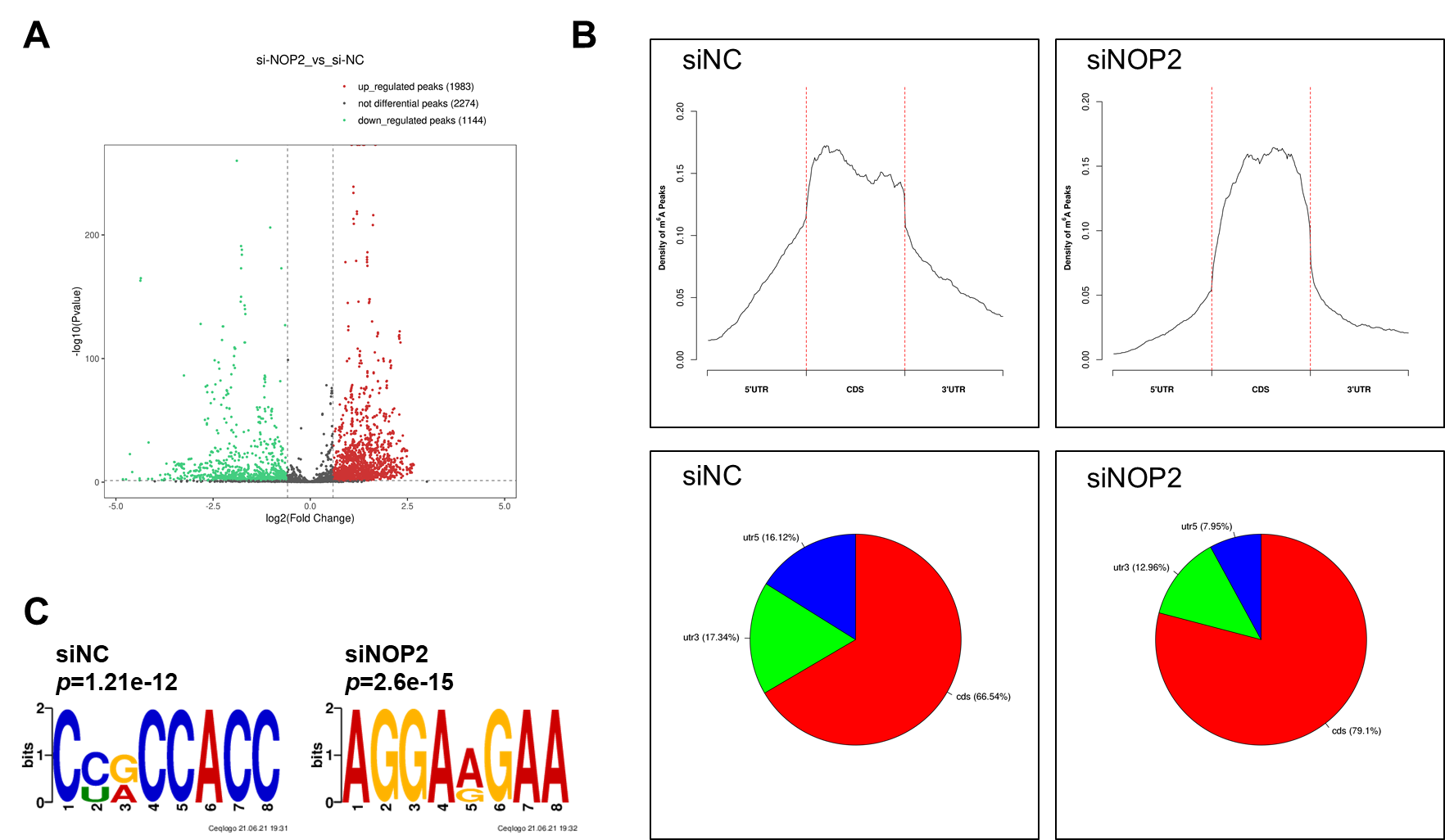


**Figure S1.** The differentially changed m5C modification peaks and the peaks distribution between siNC and siNOP2 groups. A, Volcano plot shows the differentially changed m5C modification peaks of mRNAs in NOP2-silenced cells compared with siNC cells. Red dot indicates significantly up-regulated peaks and green dot means significantly down-regulated peaks (log_2_FC>1.5, *p*<0.05). B, The peaks distribution and proportion in different region of mRNAs between siNC and siNOP2 groups. C, The most common motifs of enrichment peak in siNC group and siNOP2 group.


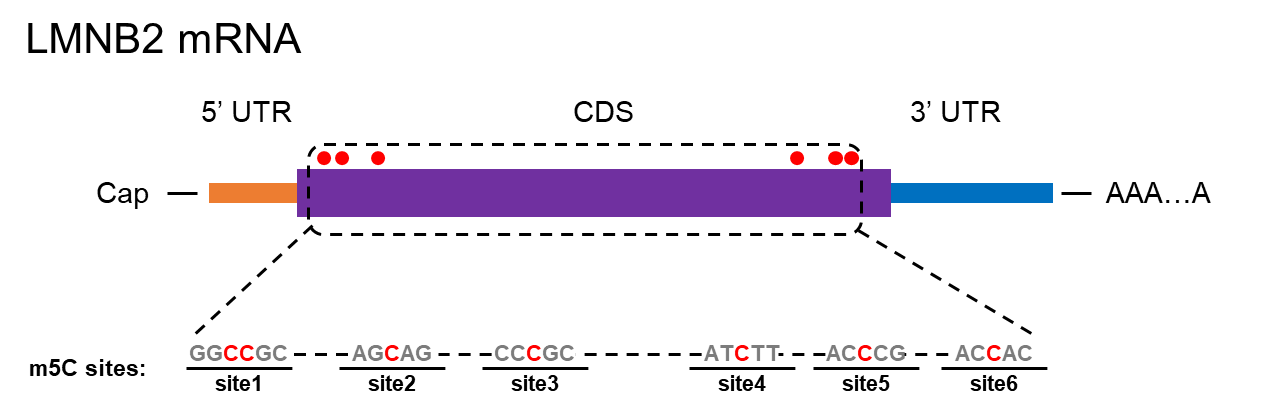


**Figure S2.** The m5C modification sites in the CDS region of LMNB2 mRNA were predicted by the online tool RNAm5Cfinder.

| **Table S1:** Information of deep-frozen CRC specimens | |  |  |
| --- | --- | --- | --- |
| **No.** | **Pathological type** | **Gender** | **Age** |
| 1 | Well-differentiated adenocarcinoma | M | 71 |
| 2 | Moderately-differentiated adenocarcinoma | M | 40 |
| 3 | Moderately-differentiated adenocarcinoma | M | 57 |
| 4 | Mucinous adenocarcinoma | M | 83 |
| 5 | Well to moderately differentiated adenocarcinoma | M | 76 |
| 6 | Mucinous adenocarcinoma | F | 73 |
| 7 | Moderately-differentiated adenocarcinoma | M | 85 |
| 8 | Moderately-differentiated adenocarcinoma | F | 66 |
| 9 | Moderately-differentiated adenocarcinoma | F | 70 |
| 10 | Mucinous adenocarcinoma | F | 77 |
| 11 | Signet ring cell carcinoma (adenocarcinoma) | M | 59 |
| 12 | Mucinous adenocarcinoma | M | 31 |
| 13 | Moderately-differentiated adenocarcinoma | F | 65 |
| 14 | Moderately-differentiated adenocarcinoma | F | 50 |
| 15 | Moderately-differentiated adenocarcinoma | M | 84 |
| 16 | Moderately-differentiated adenocarcinoma | M | 82 |
| 17 | Well-differentiated adenocarcinoma | M | 50 |
| 18 | Moderately-differentiated adenocarcinoma | M | 62 |
| 19 | Moderately-differentiated adenocarcinoma | F | 68 |
| 20 | Moderately-differentiated adenocarcinoma | M | 76 |
| 21 | Moderately-differentiated adenocarcinoma | M | 81 |
| 22 | Moderately-differentiated adenocarcinoma | M | 52 |
| 23 | Moderately-differentiated adenocarcinoma | F | 69 |
| 24 | Moderately-differentiated adenocarcinoma | F | 67 |
| 25 | Moderately-differentiated adenocarcinoma | F | 84 |
| 26 | Moderately to poorly differentiated adenocarcinoma | F | 65 |
